# Gurjun Oil in Leprosy

**Published:** 1881-07

**Authors:** 


Gurjun Oil in Leprosy.—Surgeon C. T. Peters has submitted a report which has been circulated by the Bombay Government, detailing the result of the use of gurjun oil in the Roman Catholic Leper Asylum of Belgium. Twenty-nine patients were subjected to treatment, which consisted of (1) inunction of the whole body every morning with carbolic acid (1 in 40); (2) a bath of warm water and soap a few hours afterward; (3) an application of gurjun emulsion, one to three of lime-water, to the affected and ulcerated parts; (4) application of cashew-nut oil to anæsthetic and ulcerated parts; (5) chaulmoogra oil in five-minim doses, with five grains of bicarbonate of soda, and an ounce of peppermint water internally. Under this plan considerable improvement took place, the sores were healed, tubercles absorbed, and sensibility was partially restored. Dr. Peters summarizes the merits of gurjun oil as follows: (1) Its rapidly healing action on chronic leprous ulcers. (2) It softens the skin. (3) Prevents the collection of flies. (4) Its cheapness. (5) Its efficacy in the treatment of chronic skin diseases. Surgeon-General Hunter, in forwarding the paper, however, remarks that an extended treatment by medical officers of gurjun oil in the treatment of leprosy has only ended in disappointment.—*Calcutta Medical Journal.*

